# Socioeconomic Vulnerability to Depressive Symptoms in Patients with Chronic Hepatitis B

**DOI:** 10.3390/ijerph16020255

**Published:** 2019-01-17

**Authors:** Thuc Thi Minh Vu, Thieu Van Le, Anh Kim Dang, Long Hoang Nguyen, Binh Cong Nguyen, Bach Xuan Tran, Carl A. Latkin, Cyrus S. H. Ho, Roger C. M. Ho

**Affiliations:** 1 Hanoi Medical University, Hanoi 100000, Vietnam; vuminhthuc2010@yahoo.com.vn; 2 Viet-Tiep Friendship Hospital, Hai Phong 180000, Vietnam; thieulv@gmail.com (T.V.L.); nguyencongbinhvt@gmail.com (B.C.N.); 3 Institute for Global Health Innovations, Duy Tan University, Da Nang 550000, Vietnam; kimanh.ighi@gmail.com; 4 Center of Excellence in Behavioral Medicine, Nguyen Tat Thanh University, Ho Chi Minh City 700000, Vietnam; longnh.ph@gmail.com (L.H.N.); hocmroger@yahoo.com.sg (R.C.M.H.); 5 Institute for Preventive Medicine and Public Health, Hanoi Medical University, Hanoi 100000, Vietnam; 6 Bloomberg School of Public Health, Johns Hopkins University, Baltimore, MD 21205, USA; carl.latkin@jhu.edu; 7 Department of Psychological Medicine, National University Hospital, Singapore 119074, Singapore; cyrushosh@gmail.com; 8 Department of Psychological Medicine, Yong Loo Lin School of Medicine, National University of Singapore, Singapore 119228, Singapore

**Keywords:** socioeconomic, inequality, depressive symptoms, chronic hepatitis B, Vietnam

## Abstract

Depression is considered one of the most prevalent psychiatric disorders among patients with hepatitis B virus (HBV)-related liver disease and has adverse effects on the disease progression. However, there is a scarcity of studies contributing to the assessement of depression in hepatitis B patients. There is also little research into risk factors, particularly underlying socio-economic factors in Vietnam where the prevalence of hepatitis B is high. This study aimed to examine depression and identify whether differences in socio-economic status is related to the level of depression amongst chronic hepatitis B patients. A cross-sectional study was conducted on 298 patients with chronic hepatitis B at The Chronic Hepatitis Clinic in the Viet-Tiep Hospital, Hai Phong, Vietnam. The Patient Health Questionnaire-9 (PHQ-9) and EuroQol-5 dimensions-5 levels (EQ-5D-5L) were used to assess the severity of depression and health-related quality of life (HRQOL). Of chronic hepatitis B patients, 37.5% experienced depressive symptoms and most of them suffered minimal depressive symptoms (31.4%). According to the result of the multivariate logistic regression model, we found that higher age, lower income level, unemployement, living with spouse/partners were positively associated with having depression. Furthermore, having physical health problems and lower health-related quality of life were also related to a higher risk of depression. We recommend family support, financial support and active participation in consultation should be conducted during treatment to improve the quality of life and the emotional state of HBV patients.

## 1. Introduction

Hepatitis B is regarded as a major global health issue which causes liver infection and potentially threatens the life of patients. It can progress to chronic hepatitis B and be accompanied with severe complications such as cirrhosis and liver cancer [1]. According to the report of Global Health Data Exchange in 2017, there are approximately 257 million hepatitis B patients; acute hepatitis B caused the loss of about 3,525,872 disability-adjusted life-years (DALYs) and 0.16% of the death of the population. The loss of DALYs and death regarding cirrhosis and other chronic liver diseases due to hepatitis B were the highest (12,209,930 DALYs and 0.69% respectively); the results for liver cancer were 9,547,138 DALYs and 0.58% [2]. It is estimated that 2.0% of the general population in the South-East Asia Region is infected with hepatitis B [1] and in particular, the prevalence of hepatitis B in Vietnam is high, with an estimated of 8.6 million people being infected [3].

Given the fact that chronic hepatitis B can hardly be cured and patients have to live with the disease for such a long time [4], it may strongly impact the patient’s mental status via certain psychosocial pathways [5]. Because hepatitis B virus (HBV) can be transmitted in the general population, hepatitis B patients are prone to suffer from stigma and discrimination [6,7] which may result in a negative social response [5]. Patients can feel loneliness, hopelessness and tend to be socially isolated, leading to an increased vulnerability to depression [8]. The onset of depression is also attributed to disease progression and complications such as hepatic failure and cirrhosis, in which patients may suffer from serious symptoms such as edema, ascites, fatigue or jaundice [9,10,11]. Furthermore, the development of depression can be considered a side effect of interferon therapy, which is the main agent in hepatitis treatment [12,13].

Patients with chronic hepatitis suffering from depression may be less likely to comply and follow up with medical treatment [12]. In addition, their physical cooperation and social activities involvement may decrease [14]. The prevalence of depression among hepatitis B patients also varies based on different measurements, for instance 6.4% in China in 2014 (using Mini International Neuropsychiatric Interview (MINI)) [15]; 19.8% in Iran in 2011 (measuring by Hamilton Scale) [16]; 40.6% of patients had severe depression in Turkey in 2012 using Beck Depression Inventory [17]. Socio-economic inequalities are considered significant predictors for the occurrence of depression. Specifically, one study in China indicated associations between social economic conditions such as lower household income, being male, living in rural areas and depressive symptoms [5]. Other studies revealed that living with spouse/partner could also be considered at risk of having depression due to the fear of transmission to the spouse, as well as family and social acceptance [18,19]. Age and occupations were also treated as associated factors of depression among HBV patients [5,12].

In Vietnam, empirical evidence to identify which groups of HBV patients are socially vulnerable to depression is limited. Ensuring equal treatment and care benefits among patients with different socio-economic conditions is vital to achieve optimal outcomes. This is particularly important for those at high risk of poor treatment outcomes and physical and psychological problems [20]. Therefore, our study aimed to examine the prevalence of depression in patients with chronic hepatitis B (CHB) in Vietnam and identify whether differences based on their socio-economic status relates to depression level.

## 2. Materials and Methods

### 2.1. Study Setting and Sampling Method

This study used data obtained from a cross-sectional study, which was conducted in October 2018 at the Chronic Hepatitis Clinic in the Viet-Tiep Hospital, Hai Phong, Vietnam. We included patients who were diagnosed with chronic hepatitis B (CHB), and aged 18 years old or above. We excluded patients if they had cognitive impairment or other severe health conditions that might affect their capability to answer questions in the interview. The convenient sampling method was used to recruit CHB patients attending the clinics. They were face-to-face interviewed while being examined by physicians and nurses at the clinic. The interviewers were trained intensively and repeatedly by the principal investigator to ensure the data quality. All patients participating in the study were informed and asked to give their written informed consent. A total of 298 CHB enrolled in the study.

### 2.2. Data Measurement

Data about socioeconomic, clinical and depressive symptoms were collected using a structured questionnaire. This tool was piloted in ten patients and was modified according to their comments to ensure the text and logical issue of each item. For further information about the questionnaire, please find in supplementary material 1.

#### 2.2.1. Socioeconomic and Health Status Information

Age, gender, education level, employment, marital status and household monthly income (in million vietnam dong (VND)) were obtained. We also acquired data about the number of comorbidities that were suffered. There were two items regarding pain and discomfort and anxiety and depression. The EuroQOL-5 dimensions-5 levels (EQ-5D-5L) instrument was utilized along with EQ-visual analogue scale (EQ-VAS) [21].

#### 2.2.2. Substance Use

We explored whether patients currently smoked or not by asking them to report their smoking status in the last 30 days. Moreover, Alcohol Use Disorders Identification Test-Consumption (AUDIT-C) was employed to identify alcohol use disorders among CHB patients. This instrument had a score ranging from 0 to 12 points. Patients were AUDIT-C positive if they were male and had a score of 4 or more; or they were female and had a score of 3 or more.

#### 2.2.3. Depressive Symptoms

We used the Patient Health Questionnaire (PHQ-9) to screen depressive symptoms via nine self-reported questions. This instrument was developed following the Diagnostic and Statistical Manual of Mental Disorders (DSM-IV) criteria. Each item had four option levels from “not at all” (0 point) to “nearly every day” (3 points), resulting in a total score ranging from 0 to 27 points. Patients were divided into different severity groups comprising: none (0), minimal (1–4), mild (5–9), moderate (10–14), moderately severe (15–19) and severe (20–27) [22]. The internal consistency reliability of this instrument was good, with the Cronbach’s alpha at 0.8419.

### 2.3. Statistical Analysis

Data analysis was performed using STATA version 15.0 (Stata Corp. LP, College Station, TX, USA). Descriptive statistics were used to present the socioeconomic variables and the severity of depression among participants. Chi-squared test was conducted to compare the difference in the prevalence of depressive symptoms among various socioeconomic groups. Multivariate logistic and Tobit regressions were employed to determine associated factors with “Having depressive symptoms” (Yes/No—binary variable) and “Patient Health Questionnaire (PHQ-9) score” (Censored continuous variable), respectively. Independent variables included socio-economic characteristics (age, gender, marital status, educational level, occupation, income level), having problems with pain and anxiety, AUDIT-C score and current smoking status. These models combined with stepwise backward selection strategies, with the threshold of *p*-value for variable selection of 0.2, in order to produce reduced models. *P*-value less than 0.05 indicated statistical significance.

### 2.4. Ethical Approval

This study was approved by the Institutional Review Board of Hai Phong University of Medicine and Pharmacy (128/HDDDBVHNVT).

## 3. Results

Table 1 shows the socio-economic characteristics of 298 CHB patients. There were 54.5% male patients, 81.1% of patients having high school education or more, 89.2% of patients having a spouse or partner and 36.4% of patients being freelancers. The mean age was 49.2 years old (SD = 16.0).

5.7% and 6.7% of participants had problems with pain and anxiety, respectively. There were 111 patients experiencing depressive symptoms (37.5%). Most of them suffered minimal depressive symptoms (31.4%), followed by mild depressive symptoms (5.7%). The mean PHQ-9 score was 1.0 (SD = 1.9). There were also only 4.0% and 14.4% of patients having AUDIT-C positive and current smoking status, correspondingly (Table 2).

Table 3 shows that significant differences were found among age and education groups regarding the presence of depressive symptoms (*p* < 0.05).

Multivariate regressions in Table 4 show that patients aged 31–45 years (OR = 2.18; 95% CI = 1.00–4.74), having problems with pain (OR = 32.61; 95% CI = 4.28–248.39) and a higher number of comorbidities (OR = 1.84; 95% CI = 1.17–2.88) were associated with a higher likelihood of having depressive symptoms. Meanwhile, higher income quintile, freelancer and higher EQ-VAS were negatively related to the presence of depressive symptoms.

Patients aged 31–45 (Coef. = 1.17; 95% CI = 0.12–2.22), having a spouse/partner (Coef. = 2.35; 95% CI = 0.44–4.26) and having problems with pain were positively correlated to PHQ-9 score. By contrast, higher education, higher household income, freelancer or blue-collar workers/farmers and higher EQ-VAS were negatively related to PHQ-9 score.

## 4. Discussion

Findings of this study provide valuable information regarding socioeconomic vulnerability to depression among patients suffering from hepatitis B. A high percentage of the participants had depressive symptoms. We found that a higher age was related to having depressive symptoms. By contrast, higher income level and being a freelancer were positively associated with lower risk of having depression compared to low-income level and unemployment. Moreover, a lower PHQ-9 score was also correlated with a higher education level compared to less than high school, while living with a spouse/partner was related to higher a PHQ-9 score compared to being single. Furthermore, having physical health problems and lower health-related quality of life were related to a higher risk of depression. These results are attributed to the development of interventions which aim to reduce the prevalence of depression among the hepatitis population in the future.

The prevalence of depression among participants in this study was relatively lower than the figures shown in other previous studies such as 68% in Iran, 49% in Turkey and 58.6% in Pakistan [12,17,23]. The discrepancy can be explained by the difference in the severity level of HBV progression as well as the measurement instruments for assessing depression. In addition, in our study, the percentage of patients having depressive symptoms was statistically higher than the Vietnamese general population (1.36%) [2]. Patients suffering from HBV-related diseases often faced several major physical symptoms which can increase the likelihood of having depression such as fatigue, ascites or edema as well as muscle cramps [15,24]. Moreover, depressive symptoms can be caused by perceived stigma and social isolation [25,26]. HBV patients may face discrimination at work and school, disrupted relationships as well as social ostracism due to the fear of HBV transmission [25]. Another factor that can contribute to depression development is the side effect of HBV medication which can make patients feel bad all over, depressed as well as losing their appetite [17,27].

We also found that older patients were more likely to have depression compared to younger patients. This may be because older patients have poorer immune system function and they may face more complications having be diagnosed for longer [12]. Furthermore, lower income (poorest compared to rich and richest) and unemployment (compared to freelancer and blue-collar worker) increased the risk of having depression among HBV patients. People with low income in Vietnam are more vulnerable to have chronic diseases and be exposed to risk factors [28]. Material deprivation, being under social psychological stress, low living conditions, as well as limitedof access to adequate health care systems are primary factors that may lead the poor to be easily afflicted by chronic diseases [29]. Moreover, unemployment may amplify financial burden of chronic diseases treatment.

The results suggested that a higher risk of suffering from depression was associated with living with a spouse or partner compared to those who were single. Patients with HBV can perceive that they bring trouble or shame to the family because of the fear of sharing experiences and putting other family members at a higher risk of HBV transmission [18]. They may feel shame and guilt, internalize social prejudices which may cause concern about losing the respect from their family and community [30]. Therefore, patients may avoid contact with others, the harmony of family relationships can potentially be disrupted which may result in depressive symptoms [25].

Patients who have pain may have depression with longer duration and greater severity compared to those do not [31]. Among HIV patients, especially those who are symptomatic or in AIDS stage, they may consider pain as a symptom of depression or as an aversion which triggers an extreme negative reaction such as depression [32,33]. Conversely, the depressive disorder can forecast the development of pain due to the somatization of emotional problems [34].

Furthermore, a negative association was observed between health-related quality of life score and depression and this result is consistent with a previous study [17]. Quality of life, which is defined as the status of general well-being including physical, emotional and psychological, is a holistic approach rather than other clinical assessments [35]. Since HBV is a chronic disease, the mortality does not immediately happen and patients have to live with the disease for their whole life. Therefore, their general well-being, as well as functionality play a crucial role [36]. Thus, the relationship between quality of life and depression is bi-directional, meaning that lower quality of life can lead to having depression and by contrast, depression may also negatively affect QOL (Quality of Life) in HBV patients. In term of physical health, participants who had problems with pain and had a higher number of comorbidities were more likely to have depression. Longer duration and more severe depression can occur among patients with pain [37]. On the other hand, depressive syndrome can predict the development of pain because of the somatization of emotional issues [34]. Our finding is also similar to the previous study which showed that the presence of medical comorbidities among HBV patients may increase the risk of psychiatric complications [38].

Several implications can be drawn from this study. First, support from family members, especially in regard to mental health problems should be promoted in order to reduce depression and enhance overall well-being among HBV patients. Secondly, because the fee for chronic hepatitis B treatment is expensive and must be paid long-term, HBV patient should receive financial support as well as career counseling which may help them to afford to pay for treatment. Finally, an integrated evaluation, as well as active participation in consultation should be conducted during treatment to improve the quality of life and the emotional state of HBV patients.

Some limitations should be acknowledged in this study. First, data which were collected by self-report can be influenced by recall bias, as well as social desirability. Second, we used a convenient sampling technique to recruit participants, which may affect the generalizability of the results to the study populations. The causal inferences between depression and predictors cannot be established due to the cross-sectional study design. In our study, the sample size was relatively small which may affect the statistical significance of research results.

## 5. Conclusions

In conclusion, a high percentage of chronic hepatitis B patients experienced depressive symptoms. We found a higher age was related to having depressive symptoms. By contrast, higher income level and being a freelance worker were positively associated with a lower risk of having depression compared to low-income level and unemployment. Moreover, a lower PHQ-9 score also correlated with a higher education level compared to a level less than high school, while living with a spouse or partner was related to a higher PHQ-9 score compared to being single. Family support, financial support and active participation in consultation should be conducted during treatment to improve the quality of life and the emotional state of HBV patients.

## Figures and Tables

**Table 1:** Socio-economic characteristics.

Characteristics	*n*	%
Gender (*n* = 297)		
Male	162	54.5
Female	135	45.5
Age-group (*n* = 298)		
≤30 years old	51	17.1
31–45 years old	69	23.2
46–60 years old	91	30.5
>60 years old	87	29.2
Education (*n* = 296)		
<High school	56	18.9
High school	125	42.2
>High school	115	38.9
Marital status (*n* = 297)		
Single	23	7.7
Having spouse/partner	265	89.2
Divorce/Widow	9	3
Occupation (*n* = 291)		
Unemployed	12	4.1
Freelancer	106	36.4
White-collar workers	59	20.3
Farmer/Blue-collar workers	100	34.4
Others	14	4.8
Characteristics	Mean	SD
Age	49.2	16.0
Monthly household income (million vietnam dong)	13.2	5.7

**Table 2:** Health status, behaviors and depressive symptoms according to Patient Health Questionnaire (PHQ-9).

Characteristics	*n*	%
Having problems with pain (*n* = 298)	17	5.7
Having problems with anxiety (*n* = 298)	20	6.7
Number of co-morbidities (*n* = 298)		
0	155	52.0
1	89	29.9
2	54	18.1
Severity of depressive symptoms (PHQ-9) (*n* = 296)		
No depressive symptoms	185	62.5
Minimal depressive symptoms	93	31.4
Mild depressive symptoms	17	5.7
Moderate depressive symptoms	1	0.3
AUDIT-C positive (*n* = 298)	12	4.0
Current smoking (*n* = 298)	43	14.4
Characteristics	Mean	SD
PHQ-9 score	1.0	1.9
EQ-VAS score	74.5	13.1
AUDIT-C score	0.5	1.3

**Table 3:** Depressive symptoms according to different socio-economic characteristics.

Characteristics	Having Depressive Symptoms				*p*-Value
	No		Yes		
	*n*	%	*n*	%	
Gender (*n* = 295)					
Male	96	51.9	65	59.1	0.23
Female	89	48.1	45	40.9	
Age-group (*n* = 296)					
≤30 years old	46	24.9	5	4.5	<0.01
31–45 years old	44	23.8	24	21.6	
46–60 years old	56	30.3	34	30.6	
>60 years old	39	21.1	48	43.2	
Education (*n* = 294)					
<High school	25	13.6	31	28.2	<0.01
High school	76	41.3	47	42.7	
>High school	83	45.1	32	29.1	
Marital status (*n* = 295)					
Single	18	9.7	5	4.5	0.26
Having spouse/partner	162	87.6	101	91.8	
Divorce/Widow	5	2.7	4	3.6	
Occupation (*n* = 289)					
Unemployed	6	3.3	6	5.6	0.06
Freelancer	76	42	29	26.9	
White-collar workers	36	19.9	23	21.3	
Farmer/Blue-collar workers	58	32	41	38	
Others	5	2.8	9	8.3	

**Table 4:** Associated factors with depressive symptoms in patients with chronic hepatitis B.

Characteristics	Having Depressive Symptoms		PHQ-9 Score	
	Odd Ratio (OR)	95% Confidence Interval (95% CI)	Coefficient (Coef.)	95% Confidence Interval (95% CI)
Age group				
≤30 years old	ref		ref	
31–45 years old	2.18 **	1.02; 4.74	1.17 **	0.12; 2.22
Education				
<High school			ref	
High school			−1.12 **	−2.20; −0.03
>High school			−1.64 **	−2.90; −0.37
Household income quintiles				
Poorest	ref		ref	
Rich	0.32 **	0.13; 0.76	−1.21 *	−2.47; 0.06
Richest	0.06 **	0.04; 0.86	−3.28 **	−6.40; −0.17
Occupation				
Unemployed	ref		ref	
Freelancer	0.39 ***	0.19; 0.77	−2.05 ***	−3.23; −0.87
Farmer/Blue-collar workers			−1.28 **	−2.35; −0.20
Marital status				
Single	ref		ref	
Having spouse/partner	3.66 *	0.95; 14.13	2.35 **	0.44; 4.26
Having problems with anxiety				
No	ref		ref	
Yes	0.22	0.03; 1.46		
Having problems with pain				
No	ref		ref	
Yes	32.61 ***	4.28; 248.39	3.62 ***	1.98; 5.27
EQ-VAS	0.96 ***	0.93; 0.99	−0.10 ***	−0.14; −0.06
Number of comorbidities	1.84 ***	1.17; 2.88		
AUDIT-C score			−0.24	−0.60; 0.11

* *p* < 0.05; ** *p* < 0.01; *** *p* < 0.001. Ref: reference.

## Supplementary Materials

The following are available online at http://www.mdpi.com/1660-4601/16/2/255/s1, supplementary material 1: Survey of mental health status of patients having chronic liver B disease at Viet Tiep Hai Phong Friendship Hospital.
