# Supplementary material for: Socioeconomic Vulnerability to Depressive Symptoms in Patients with Chronic Hepatitis B
Source: Int J Environ Res Public Health. 2019 Jan 17;16(2):255. doi: 10.3390/ijerph16020255 (PMC6352008; doi:10.3390/ijerph16020255)
Supplement: Supplementary file 1 [file ijerph-16-00255-s001.pdf]

Supplementary Material 1. Survey of mental health status of patients having chronic liver B disease at Viet Tiep Hai Phong Friendship Hospital.

A	**General information**	
**A1.**	**Year of birth?**	…………………
**A2.**	**Gender?**	
	Male	1
	Female	2
**A3.**	**Highest educational level?**	
	Illiteracy	1
	Primary school	2
	Secondary school	3
	High school	4
	College, vocational training	5
	University	6
	Post-graduated	7
**A4.**	**Marital status?**	
	Single	1
	Living with your spouse/partner	2
	Living together as a spouse, unmarried	3
	Divorce / Separation	4
	Widow	5
**A5.**	**Current occupation? (with income)**	
	Unemployment	1
	Freelance	2
	White-collar worker	3
	Blue-collar worker, Farmers	4
	Student	5
	Other occupations (with income)	6
**A6.**	**Within the last year, what is the average monthly income of your family?**	………….. VND
B	**Health status**	
**B1**	Next, I would like to ask you 5 questions about the health status today. Please answer based on the scale of 1 to 5. In which 1 is "being unable/extremely" and 5 is "No problems".	

Today…….	**Being unable**	**Severe proble ms**	**Moderate problems**	**Slight proble ms**	**No proble ms**
1. Do you have problems in walking about?	1	2	3	4	5
2. Do you have problems in washing or dressing yourself?	1	2	3	4	5
3. Do you have problems in doing your usual activities ?	1	2	3	4	5

	**Extreme**	**Severe**	**Moderat e**	**Slight**	**No proble m**
4. Do you have pain or discomfort?	1	2	3	4	5
5. Do you have anxiety or depression	1	2	3	4	5

**B4**	**Do you have any health problems (diseases or symptoms)?**

1.	4.	7.
2.	5.	8.
3.	6.	9.

B5 Next, please answer 9 questions about some issues in the past 2 weeks. Please circle the appropriate answer in each sentence!

**Over the last two weeks, how often have you been bothered by any of the following problems?**	*Circle the corresponding option*			
	**Not at all**	**Severa l days**	**More than half of the days**	**Nearly everyday**
Little interest or pleasure in doing thing	0	1	2	3
Feeling down, depressed, or hopeless	0	1	2	3
Trouble falling or staying asleep, or sleeping too much	0	1	2	3
Feeling tired or having little energy	0	1	2	3
Poor appetite or overeating	0	1	2	3
Feeling bad about yourself - or that you are a failure or have let yourself or your family down	0	1	2	3
Trouble concentrating on things, such as reading the newspaper or watching television	0	1	2	3
Moving or speaking so slowly that other people could have noticed. Or the opposite - being so fidgety or restless that you have been moving around a lot more than usual	0	1	2	3
Thoughts that you would be better off dead, or of hurting yourself in some way	0	1	2	3

**C**	**Health risk behavior**	
**C1**	**In recent times, how often do you drink alcohol?**	
	Never	1
	Monthly	2
	Weekly	3
	2-3 times per week	4
	> = 4 times per week	5
**C2**	**Usually when drinking alcohol, how often do you drink cups?**	………………cups
**C3**	**How often do you drink 6 or more cups of alcohol?**	
	Never	1
	Every few months	2
	Monthly	3
	Weekly	4
	Daily	5
**C4**	**Within the last 30 days, did you smoke cigarettes / pipe tobacco?**	
	Yes, smoke cigarette	1
	Yes, smoking pipe tobacco	2
	Yes, smoking both types	3
	No	4
